# Admission lactate and outcome after high-risk surgery

**DOI:** 10.1186/cc10868

**Published:** 2012-03-20

**Authors:** M Geisen, HD Aya, C Ebm, N Arulkumaran, MA Hamilton, M Grounds, A Rhodes, M Cecconi

**Affiliations:** 1 St George's Hospital NHS Trust, London, UK

## Introduction

The aim of this study was to assess the ability of serum lactate level in patients admitted to the ICU after surgery to predict outcome.

## Methods

A retrospective, clinical observational study in patients undergoing high-risk surgery admitted to a 17-bed ICU of a large teaching hospital. Data were obtained during haemodynamic optimization using an established GDT protocol in the first 8 hours after admission and included demographic data as well as haemodynamic and laboratory parameters. Outcome data included morbidity (defined as >1 complications on the postoperative morbidity survey) and clinical outcome (hospital mortality, length of ICU stay, length of hospital stay, readmission to the ICU).

## Results

**Figure 1:**
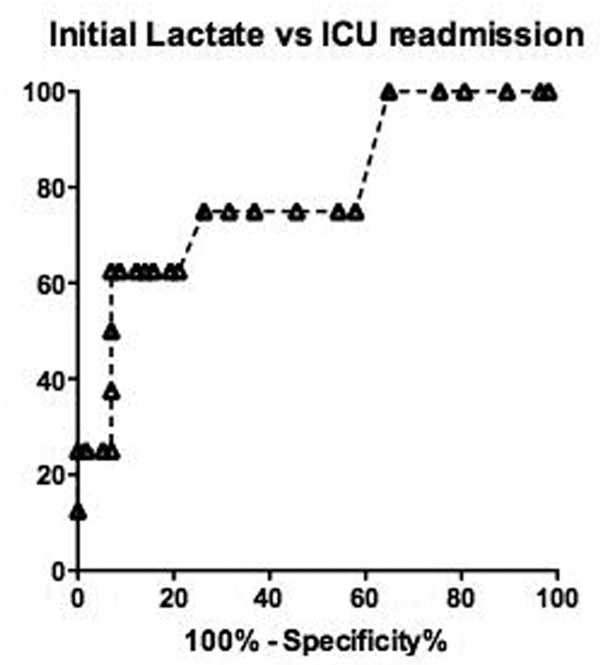
**Prediction of ICU readmission according to initial lactate concentration**.

Sixty-seven patients were included. Lactate clearance (decrease of lactate >10% in 2 hours) occurred in 64 patients (96%). Sixty patients developed at least one surgical complication. There were no significant correlation between lactate levels on admission and development of complications and length of hospital stay. Nine patients (13%) were readmitted to the ICU. A receiving operator characteristic analysis for readmission to the ICU showed an area under the curve of 0.79. A lactate higher than 1.7 mmol/l on admission had a sensitivity of 75% and a specificity of 74% to predict ICU readmission (Figure 1). Patients with a lactate on admission >1.7 mmol/l also had a longer length of ICU stay (Table 1).

**Table 1:** Lactate on admission, complications and clinical outcome

	Lactate <1.7 mmol (*n *= 46)	Lactate >1.7 mmol (*n *= 21)	*P *value
Complications	41 (89%)	19 (90.5%)	NS
Complications >1	36 (78.3%)	16 (76.2%)	NS
Total complications per patient	3 (2 to 7)	4 (2 to 7)	NS
Hospital stay	14 (8 to 39)	13 (8 to 24)	NS
ICU stay	1 (1 to 2)	2 (1 to 10)	0.045
Readmission to ICU	3 (6.5%)	6 (28.6%)	0.022
Mortality	2 (4.3%)	3 (14.3%)	NS

## Conclusion

Lactate on admission correlates with length of ICU stay and readmission to the ICU.
